# Sepsis: Pathogenesis, Biomarkers, and Treatment

**DOI:** 10.1155/2015/846935

**Published:** 2015-03-05

**Authors:** Baoli Cheng, Andreas H. Hoeft, Malte Book, Qiang Shu, Stephen M. Pastores

**Affiliations:** ^1^Department of Anesthesiology, The First Affiliated Hospital, School of Medicine, Zhejiang University, Qingchun Road 79, Hangzhou 310003, China; ^2^Department of Anesthesiology and Intensive Care Medicine, University Hospital Bonn, Bonn, Germany; ^3^Department of Anesthesiology, Bern University Hospital, 3010 Bern, Switzerland; ^4^Department of Surgery, Children's Hospital, School of Medicine, Zhejiang University, Zhejiang, China; ^5^Memorial Sloan-Kettering Cancer Center, New York, NY, USA

Sepsis is an infection-initiated systemic inflammatory syndrome with an estimated incidence of 18 million cases annually worldwide. Despite advances in intensive care and supportive technology, the mortality rate of sepsis still ranges from 15% to 80%, reminding scientists and clinicians that it remains to be a major clinical challenge. The key to winning the “campaign” to combat sepsis is improved understanding of the epidemiology, pathogenesis, and biomarkers of sepsis and discovery of novel therapies. The present special issue shows several encouraging results and provides comprehensive reviews of the latest advances in this field.

The effector cells from the innate and adaptive immune systems play a crucial role in sepsis. Dendritic cells, in particular, serve as professional antigen presenting cells and are involved in the aberrant immune response to sepsis. In this special issue, X. Fan et al. discuss the effects of sepsis on the amount, surface molecule expression, cytokine secretion, and T-cell activating function of dendritic cells and the underlying mechanisms in their review “Alterations of Dendritic Cells in Sepsis: Featured Role in Immunoparalysis.” Recent postmortem studies of patients who died of sepsis showed that depletion of CD4 and CD8 lymphocytes is an important characteristic. Thus, knowledge of these circulating lymphocyte abnormalities is relevant for the understanding of sepsis pathophysiology. R. de Pablo et al., who have previously reported on the alteration of B cells, natural killer cells, and T-cell function in septic patients, summarize their latest findings on the role of blood lymphocytes in sepsis and discuss the different kinetic patterns of lymphocyte subsets and their relationship to outcome in their review “Role of Circulating Lymphocytes in Patients with Sepsis.” Both the clinical and basic researches have shown that sepsis-associated immunosuppression is associated with adverse outcomes. A novel heterogeneous population of immature myeloid cells that possess immunosuppressive activities, termed myeloid-derivedsuppressorcells(MDSCs), has gainedmuchattention in recent sepsis studies. D. Lai et al. discuss the complex functions of MDSCs in the pathogenesis of sepsis. Their review “Myeloid-Derived Suppressor Cells in Sepsis” also proposes that the overall role of MDSCs involves much more than simply being an immunosuppressive cell population. These 3 review articles provide a comprehensive analysis of the major important immune cells in sepsis and highlight potential therapeutic targets. As a group who have investigated the function of the family of defensins in sepsis for nearly 10 years, G.-H. Xie et al. summarize the* in vitro*,* in vivo*, and genetic studies on the effects of defensins as well as the corresponding mechanisms within sepsis. Their review, “Defensins and Sepsis,” also points out that the function of defensins reflects both their immunomodulatory and broad-spectrum antimicrobial effects.

Although the international Surviving Sepsis Campaign guidelines have been released for 10 years, sepsis remains a fatal syndrome due to the lack of efficient biomarkers and novel treatments. D. N. Nguyen et al. investigated plasma cortisol levels in septic patients with delirium and coma and found that cortisol is a potential biomarker of brain dysfunction in their article “Cortisol Is an Associated-Risk Factor of Brain Dysfunction in Patients with Severe Sepsis and Septic Shock.” F. Song et al. and P. Madhusudan et al. discuss two important but controversial issues related to the Surviving Sepsis Campaign Guidelines. In a meta-analysis of 12 randomized trials involving 4100 septic patients, “Intensive Insulin Therapy for Septic Patients: A Meta-Analysis of Randomized Controlled Trials,” F. Song et al. reported no benefit and a higher incidence of hypoglycemia with intensive insulin therapy compared with conservative glucose management. P. Madhusudan et al. discuss the current debate on the choice, amount, and end points for fluid resuscitation in sepsis in their review “Fluid Resuscitation in Sepsis: Reexamining the Paradigm.” K. Xie et al. investigated the therapeutic function of hydrogen gas in a septic animal model for several years, and, in their present review, “Hydrogen Gas Presents a Promising Therapeutic Strategy for Sepsis,” they summarize the progress of hydrogen treatment in sepsis. J. Zhou et al. and X. Li et al. explore novel drugs for sepsis from the perspective of the neuroendocrine network in sepsis in their two studies, “Epinephrine Enhances the Response of Macrophages under LPS Stimulation” and “Agmatine Protects against Zymosan-Induced Acute Lung Injury in Mice by Inhibiting NF-*κ*B-Mediated Inflammatory Response.”

In this present special issue about the pathogenesis, biomarkers, and treatment of sepsis, the authors provide comprehensive reviews and attractive research perspectives on the mechanisms of sepsis which we hope will inspire researchers investigating novel biomarkers and therapeutic sepsis targets.

